# Estimated age of amyloid plaque onset and impact of APOE4 in longitudinally assessed presenilin 1 E280A autosomal dominant Alzheimer’s disease mutation carriers

**DOI:** 10.1002/alz70861_108116

**Published:** 2025-12-23

**Authors:** Valentina Ghisays, Yi Su, Shan Li, Hillary D. Protas, Yinghua Chen, Ji Luo, Javad Sohankar, Alejandro Espinosa, Claudia Aponte, Alejandro Guerrero, Ana Y Baena, Marisol Londoño Castaño, Margarita M. Giraldo‐Chica, Natalia Acosta‐Baena, Yamile Bocanegra, Eliana Henao, Silvia Ríos‐Romenets, Claudia Muñoz, Laura Serna, Eugenia Cardona, Yudy M Leon‐Varela, Paula Ospina, Victoria Tirado, Juan F Martínez, Sindy Duque, Sergio Alvarez, Tobias Bittner, Jessica B. Langbaum, Kewei Chen, Pierre N. Tariot, David Aguillon, Francisco Lopera, Yakeel T. Quiroz, Sterling C. Johnson, Eric M. Reiman, Robert C. Alexander, API ADAD Colombia Trial Group

**Affiliations:** ^1^ Banner Alzheimer's Institute, Phoenix, AZ USA; ^2^ Grupo de Neurociencias de Antioquia, Facultad de Medicina, Universidad de Antioquia, Medellín, Antioquia Colombia; ^3^ Grupo de Neurociencias de Antioquia, Facultad de Medicina, Universidad de Antioquia, Medellín Colombia; ^4^ Hospital Pablo Tobón Uribe, Medellín, Antioquia Colombia; ^5^ F. Hoffmann‐LaRoche AG, Basel Switzerland and Genentech Inc. a member of the Roche Group, South San Francisco, CA USA; ^6^ Massachusetts General Hospital, Harvard Medical School, Boston, MA USA; ^7^ University of Wisconsin School of Medicine and Public Health, University of Wisconsin‐Madison, Madison, WI USA

## Abstract

**Background:**

We previously estimated the age of amyloid plaque onset at 28.2 years using florbetapir PET measurements of cerebral amyloid deposition in a cross‐sectional study of a convenience sample of PSEN1 E280A carriers and non‐carriers, ages 20‐56, recruited from the world's largest autosomal dominant Alzheimer’s disease (ADAD) cohort. Here we capitalized on longitudinal data from a different sample than used previously, to estimate the age of amyloid onset (EOA) in carriers who were cognitively unimpaired at baseline and examine differences associated with apolipoprotein ε4 (APOE4) status and between men and women.

**Method:**

We analyzed baseline, 2‐year, and 5‐year florbetapir PET scans from 161 initially cognitively unimpairedPSEN1 E280A carriers (ages 30–56) enrolled in the Alzheimer’s Prevention Initiative (API) ADAD Colombia Trial of crenezumab vs placebo, in which crenezumab did not lower brain amyloid (NCT01998841). We included PSEN1 E280A carriers with at least 1 amyloid‐positive (A+) PET scan, using cortical‐to‐pontine standard‐uptake value ratios converted to centiloids (CL). Out of the 169 PSEN1 E280A carriers in the trial, 12 were A‐ and 149 were A+ at baseline, we excluded 1 PSEN1 E280A carrier who did not have A+ longitudinal data and 7 who did not have A+ scans at baseline or follow‐up. The Sample Iterative Local Approximation (SILA) model was applied to estimate EOA using a 20 CL threshold for positivity. We estimated amyloid chronicity and compared EOAs, adjusted for baseline age, between A+ male (*n* =68) and female (*n* =98) PSEN1 E280A carriers and between A+ PSEN1 E280A APOE4‐carriers (*n* =35) and non‐carriers (*n* =126).

**Result:**

Median EOA was 26.1 years (SD ±7.89, range 1‐54) and estimates were normally distributed. PSEN1 E280A females had a mean EOA of 25.8 years which was not significantly different than the estimates in males (M=27.8 years). PSEN1 E280A APOE4 carriers had an EOA about 3 years younger (M=24.1) than APOE4 non‐carriers (M=27.3, *p* =0.03).

**Conclusion:**

This study illustrates the ability to use longitudinal biomarker data to characterize the onset of biomarker changes and impact of putative disease modifiers like APOE4 in ADAD mutation carriers.

